# A comparative study of hourly and daily relationships between selected meteorological parameters and airborne fungal spore composition

**DOI:** 10.1007/s10453-017-9493-3

**Published:** 2017-07-19

**Authors:** Agnieszka Grinn-Gofroń, Beata Bosiacka, Aleksandra Bednarz, Tomasz Wolski

**Affiliations:** 1 Department of Plant Taxonomy and Phytogeography, Faculty of Biology, University of Szczecin, Wąska 13, 71-415 Szczecin, Poland; 2 Faculty of Geosciences, University of Szczecin, Mickiewicza 18, 70-383 Szczecin, Poland

**Keywords:** Airspora occurrence, Meteorological conditions, Hourly and daily data, Redundancy analysis (RDA)

## Abstract

Air sampling was conducted in Szczecin (Poland) throughout April–September 2013. The final data set included 177 daily and 4248 hourly samples. The total of 21 types of spores, which occurred in a number >10 in the season, were taken into account. The following meteorological parameters were analyzed: air temperature, relative humidity, precipitation and wind speed. Effects of individual weather parameters on hourly and daily concentrations of different fungal spore types were examined using Spearman’s rank association test, whereas effects of complex of meteorological factors on hourly and daily compositions of spore were assessed using detrended correspondence analysis (DCA) and redundancy analysis (RDA). Airborne fungal spore distribution patterns in relation to meteorological variables were determined by RDA, after DCA results detected a linear structure of the spore data. The RDA results obtained indicated that all the applied variables accounted for 20 and 22% of the total variance in the hourly and daily spore data, respectively. The results of stepwise forward selection of variables revealed all included hourly and daily meteorological variables were statistically significant. The largest amount of the total variance in the spore composition was explained by the air temperature in both cases (16%). Multivariate ordination did not show large differences between the hourly and daily relationships (with exception of wind speed impact), while the differences between simple hourly and daily correlations were more clear. Correlations between daily values of variables were in most cases higher than between hourly values of variables.

## Introduction

The spores of many fungi are displaced from their parent colonies by passive mechanisms—by physical disturbance resulting from airflow, raindrops, vibration, or by the specific vectors (e.g., insects and birds). Active mechanisms of spore discharge are powered by hydrostatic pressure, fast movements induced by cytoplasmic dehydration, and by the utilization of surface tension force. The active mechanisms of spore discharge are depended on the taxonomical group. In cases of Ascomycota, the ascus is essentially an explosive sporangium—the asci are pressurized osmotically and spores and droplets of epiplasmic fluid are vigorously ejected. Most Basidiomycota utilize the process of ballistospore discharge, which is powered by the rapid motion of a fluid droplet over the spore surface (Ingold 1971, 1999; Pringle et al. 2005).

The function of spore discharge is to free spores, which may subsequently be dispersed. Most of the spores appears in the air, where they will remain for a longer or shorter period of time and are moved to different distances. When spores are puffed and splashed from the surfaces of their colonies by rain water drops, the impact of raindrops exerts much larger forces on colonies than wind disturbance. Spores can be thrown over short distances or can be carried over longer distances by wind as free spores or associated with water droplets. Dispersal can occur a distance from a few centimeters to hundreds of kilometers, even between continents (Ingold 1971; Carlile et al. 2001).

Spore production, release and spread are elements of the whole life cycle of fungi, determined by inherent characteristic of the fungi, availability of substrates, temperature, moisture (from rain and humidity) and other environmental factors. Many fungi are mesophilic. Sporulation process takes place in a narrower range of temperatures than vegetative growth. Moreover, changes in temperature over short time intervals can affect the degree of thermal turbulence, which simultaneously dilute spore concentrations at ground level, and cause the release of more spores by mechanical disturbance (Ingold 1971; Money 2015).

The growth of most fungi is favoured by high moisture, and active mechanisms of spore discharge are also related to the availability of water. Many species of Ascomycota and Basidiomycota are actively wet spore discharging fungi. At high relative humidity, they activate discharge mechanism by hygroscopic uptake of water vapor. Dry spore discharging fungi release spores by the flow of air or by hygroscopic twisting movement, which occurs upon drying. Such spores are mostly emitted when dry, warm and windy conditions prevail (Meredith 1963; Lacey 1986; Elbert et al. 2007).

The impact of precipitation on the spore content in the air may be twofold: on the one hand raindrops may cause release of fungal spores; on the other hand the rain can remove fungal spores by rain-out and wash-out effects (Ingold 1971; Lacey 1986).

As a consequence of the types of factors affecting the release of spores and their further spread, spore occurrence in the air is determined by, among others, several interacting meteorological factors. It is important and useful (e.g., public health, agriculture, horticulture and forestry, aerobiology) to know how changes in the spore concentration and composition in the air come with changes in weather conditions, including complex of variables. Such complex interactions have been analyzed in a few studies, which considered only daily values of variables (Hjelmroos 1993; Li and Kendrick 1994, 1995; Grinn-Gofroń and Bosiacka 2015; Sadyś et al. 2015).

The aim of this study was to analyze and compare the impact of meteorological parameters on daily and hourly concentrations and compositions of fungal spores during 1 year of the study, and to pinpoint the most crucial weather parameters that are useful to describe a daily and hourly compositions of fungal spores in the air.

## Materials and methods

Aerobiological study was carried out in Szczecin when the occurrence of spores in the air was the highest (from 1 April to 30 September) in 2013. The 6-day gaps in the data set resulted from technical problems (power outage) during taking samples. Sampling took place by volumetric method (Hirst 1952) according to the recommendations of the British Aerobiology Federation (1995) and preparation and evaluation of samples based on Frenguelli (2003). Both methods were described in detail in Grinn-Gofroń and Bosiacka (2015).

We took into account 21 types of spores, which occurred in a number <10 in the season, and that we were able to identify at the level of genus (type): *Agrocybe*, *Alternaria*, *Chaetomium*, *Cladosporium*, *Coprinus*, *Curvularia*, *Didymella*, *Drechslera* type, *Epicoccum*, *Fusarium*, *Ganoderma*, *Leptosphaeria* type, *Periconia*, *Phaeosphaeria*, *Pithomyces*, *Pleospora*, *Polythrincium*, *Stachybotrys*, *Stemphylium*, *Tilletia*, *Torula.*

The meteorological parameters taken into consideration in assessment of the effect of meteorological conditions on the airborne fungal spores were: TEMP—hourly air temperature and daily mean temperature (°C), RH—hourly relative humidity and daily mean relative humidity (%), PRECIP—hourly and daily amount of precipitation (mm), WIND—hourly wind velocity and daily mean wind velocity (m s^−1^).

Minimum, maximum and mean values of selected meteorological parameters during the sampling period (01.04.–30.09.2013) in Szczecin are presented in Table 1.

**Table 1:** Values of selected meteorological parameters during the sampling period (01.04.–30. 09.2013) in Szczecin

Meteorological parameters	Min. value	Max. value	Mean value
Air temperature (°C)	−2	38	18.5
Relative humidity (%)	9	100	72.6
Precipitation (mm)	0	24	0.2
Wind velocity (m s^−1^)	0	4	0.1

### Data analysis

Normality of the variables was tested with the commonly used Kolmogorov–Smirnov and Chi-square tests. Since most of the data did not fit the normal distribution (*p* < 0.05), the Spearman’s rank association test was applied to examine effects of selected weather parameters on hourly and daily concentrations of selected airborne fungal spore (STATISTICA StatSoft v. 10.0). Effects of complex of weather parameters on hourly and daily composition of fungal spore were assessed using the software package CANOCO v. 4.5 (ter Braak and Šmilauer 2002), after log transformation of spore and meteorological data by a modified formula available in CANOCO: *Y*_ki_^*^ = log(*Ay*_*ki*_ + *B*), where *y*_*ki*_ is the concentration of *k* spores in *i* sample; the coefficients *A* and *B* are standard set as 1. Airborne fungal spore distribution patterns in relation to meteorological variables were determined by multivariate redundancy analysis (RDA), after detrended correspondence analysis (DCA) results detected a linear structure of the spore data. Detailed information about the applied multivariate methods, stepwise forward selection and tests of significance is available in Grinn-Gofroń and Bosiacka (2015).

Redundancy among the meteorological variables was explored with the variance inflation factor (VIF). VIF analysis (available in CANOCO) is a diagnostic tool used to identify useless constrains. The large value of variance inflation factor (VIF > 20) mean that a given variable is so strongly correlated with others, that the rate of the canonical variable is unstable and not suitable to interpret.

## Results

**Fig. 1:**
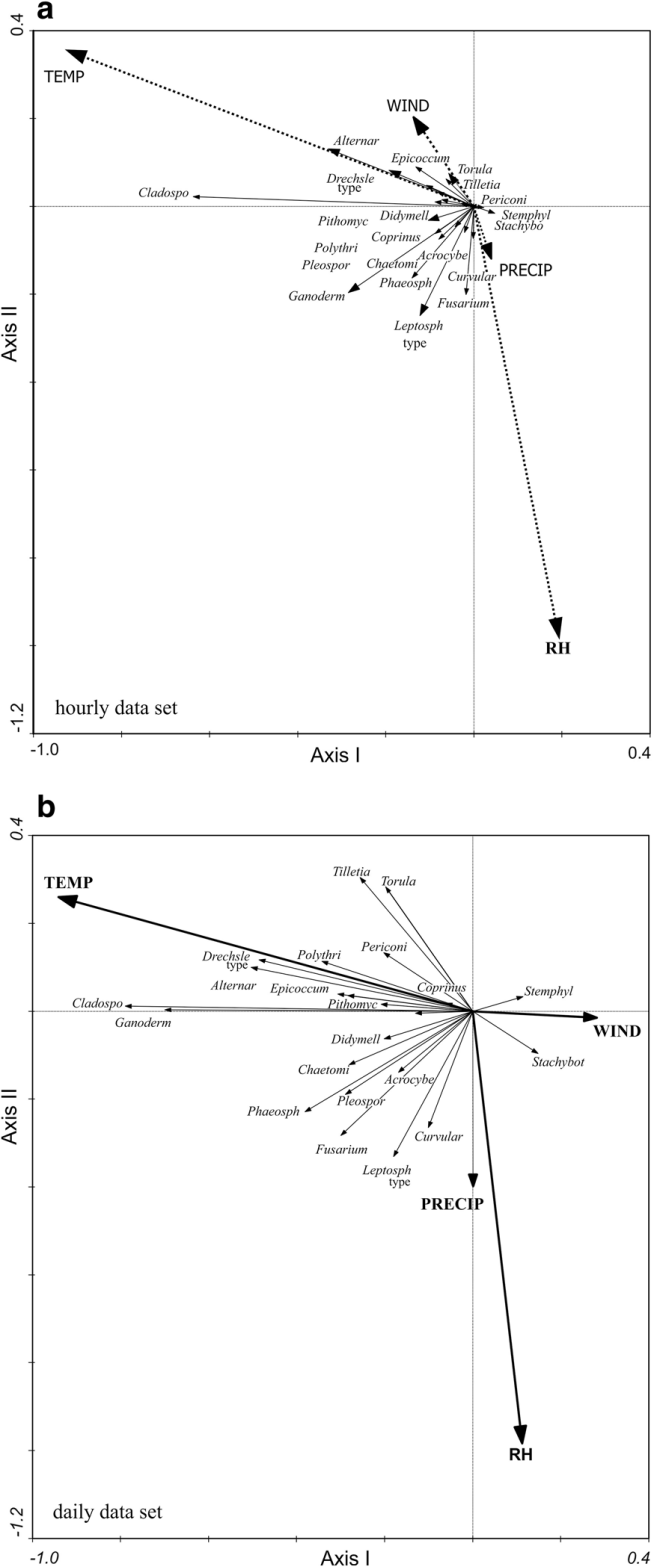
Diagrams of fungal spores and meteorological variables ordination along the first two RDA axes for hourly (**a**) and daily (**b**) samples collected in Szczecin (NW Poland)

The results of Spearman’s rank association test (Table 2) clearly showed the impact of individual meteorological parameters on the concentration of different spore types and they are supplementary to the results computed by multivariate analysis RDA provides both a comprehensive assessment of the impact of complex of meteorological parameters on specific spore composition in the air, as well as illustrates the correlation between the occurrence of particular spore types and meteorological variables. Individual dependences, visible on the RDA diagrams (Fig. 1a, b), generally corresponded to the strength and direction of the correlation in both types of analyses. However, multivariate ordination does not show large differences between the hourly and daily relationships, while the differences between simple hourly and daily correlations were more clear. Correlations between daily values of variables were in most cases higher than between hourly values of variables. The hourly relationships, due to the much higher number of samples (4248 hourly samples and 177 daily samples), were statistically significant in a larger number of cases (even very weak correlations were statistically significant).

**Table 2:** Contribution of spores and the results of Spearman’s rank correlation test between hourly and daily concentrations of fungal spores and meteorological parameters

Taxon	Seasonal contribution		Temp		RH		Wind		Precip	
	Count	%	Hourly	Daily	Hourly	Daily	Hourly	Daily	Hourly	Daily
*Agrocybe*	28	0.005	0.005	0.071	0.063*	0.165*	0.029	−0.224*	−0.024	−0.080
*Alternaria*	8648	1.586	0.359*	0.480*	−0.100*	−0.093	0.091*	−0.152	−0.078*	−0.200*
*Chaetomium*	108	0.020	0.049*	0.215*	0.085*	0.152	−0.026	−0.288*	−0.030	−0.048
*Cladosporium*	346135	63,493	0.511*	0.601*	−0.075*	−0.072	0.121*	−0.152	−0.070*	−0.084
*Coprinus*	22	0.004	0.006	0.069	−0.013	−0.066	−0.012	−0.026	0.018	0.068
*Curvularia*	19	0.003	−0.009	−0.020	0.047*	0.262*	0.008	−0.123	0.041*	0.166*
*Didymella*	175,316	32,159	0.069*	0.268*	0.057*	−0.012	0.025	−0.072	0.002	0.051
*Drechslera* type	856	0.157	0.218*	0.525*	−0.099*	−0.172*	0.022	−0.066	−0.021	−0.106
*Epicoccum*	1413	0.260	0.162*	0.169*	−0.077*	−0.038	0.033*	−0.205*	−0.042*	−0.112
*Fusarium*	30	0.006	−0.083*	0.046	0.181*	0.294*	0.064*	−0.081	0.094*	0.205*
*Ganoderma*	3586	0.658	0.179*	0.529*	0.183*	−0.086	−0.032	−0.325*	−0.027	−0.201*
*Leptosphaeria* type	7112	1.305	0.110*	0.138	0.198*	0.130	−0.026	0.125	−0.025	0.416*
*Periconia*	28	0.005	0.086*	0.182*	−0.057*	−0.035	0.047*	0.014	−0.027	0.010
*Phaeosphaeria*	448	0.082	0.052*	0.082	0.164*	0.248*	0.005	−0.179	0.035*	0.205*
*Pithomyces*	76	0.014	0.074*	0.139	−0.026	−0.037	0.025	−0.078	−0.019	0.006
*Pleospora*	715	0.131	0.083*	0.217*	−0.011	0.183*	0.044*	−0.065	0.001	0.164*
*Polythrincium*	31	0.006	0.119*	0.398*	−0.066*	−0.146	0.037*	−0.117	−0.040*	−0.185*
*Stachybotrys*	11	0.001	−0.052*	−0.215*	0.020	0.138	−0.014	−0.056	0.022	0.025
*Stemphylium*	77	0.014	−0.037*	−0.095	0.011	−0.013	0.019	−0.015	0.019	0.300*
*Tilletia*	33	0.006	0.107*	0.321*	−0.066*	−0.350*	−0.031	−0.017	0.002	−0.210*
*Torula*	463	0.085	0.098*	0.301*	−0.083*	−0.400*	−0.016	0.031	−0.029	−0.194*

* *p* < 0.05

After removing the dew point temperature (DP) from the data set (as they VIF scored above 400), the other meteorological variables did not show autocorrelation (Table 3).

**Table 3:** Redundancy among the meteorological variables with and without dew point temperatute

Variables	(Weighted) mean		Standard deviation		Variance inflation factor	
TEMP	10.4237	15.2479*	4.6059	5.1429*	392.7994	1.2262*
RH	74.0541	74.0541*	11.3110	11.3110*	102.4877	1.3386*
WIND	2.8399	2.8399*	1.0518	1.0518*	1.1336	1.1064*
PRECIP	2.3551	2.3551*	5.9782	5.9782*	1.2119	1.2105*
DP	15.2479	–	5.1429	–	457.9960	–

* Values without dew point temperature

Indirect ordination analyses (DCA) was made to check the data structure. DCA results revealed that the gradient length represented by the first ordination axis was lower than 3 SD (standard deviation), both in hourly and daily data set (2.790 and 1.470, respectively). This corresponds to a linear data structure, therefore the direct redundancy analyses (RDA) were used to determine functional relationship between composition of airspora and meteorological factors. The RDA results obtained indicated that all the applied variables (excluding DP) accounted for 20 and 22% of the total variance in the hourly and daily spore data, respectively (Table 4). First axis and all canonical axes were significant in both cases as tested by the unrestricted Monte Carlo permutation test (*p* = 0.002).

**Table 4:** Summary of RDA for hourly and daily samples

Axis	Hourly	Daily
Eigenvalues		
I	0.181	0.176
II	0.017	0.031
III	0.001	0.014
IV	0.000	0.003
Spores–environment correlations		
I	0.628	0.734
II	0.404	0.547
III	0.139	0.358
IV	0.063	0.313
Cumulative percentage variance of spore data		
I	18.1	17.6
II	19.8	20.8
III	20.0	22.1
IV	20.0	22.4
Cumulative percentage variance of spores–environment relationship		
I	90.4	90.4
II	99.2	99.2
III	99.9	99.9
IV	100.0	100.0
Sum of all eigenvalues/total inertia	1.000	1.000
Sum of all canonical eigenvalues	0.200	0.224
Percentage of explained spore data variance	20.0	22.4

The results of stepwise forward selection of variables revealed all included hourly and daily meteorological variables (TEMP, RH, PRECIP and WIND) were statistically significant (*p* ≤ 0.05). The largest amount of the total variance in the spore composition was explained by the TEMP in both cases (16%) (Table 5).

**Table 5:** Forward selection results with the test of variable significance for hourly and daily samples

Variables	Lambda A		Explained data variance (%)		F-ratio		*p* value	
	Hourly	Daily	Hourly	Daily	Hourly	Daily	Hourly	Daily
TEMP	0.16	0.16	16	16	650.42	27.34	0.002	0.002
RH	0.03	0.04	3	4	167.29	7.56	0.002	0.002
WIND	0.01	0.02	1	2	19.97	3.06	0.002	0.002
PRECIP	0.00	0.00	0.0	0.0	5.29	1.21	0.004	0.004

*p* ≤ 0.05 (significance level)

Distribution of particular fungal spore types along meteorological gradients differed only in detail between hourly and daily data (Fig. 1a, b). Following the gradient of increasing temperature of the air, the maximum abundance of *Cladosporium* spores (hourly data), and additionally *Ganoderma* spores (daily data) was related to the highest TEMP values, whereas the occurrence of spores of *Phaeosphaeria*, *Agrocybe*, *Leptosphaeria* type, *Fusarium* (hourly data) and additionally *Curvularia*, *Stachybotrys* and *Stemphylium* (daily data)—to the lowest values. Other fungal spores occurred most numerous at moderate values of the air temperature.

Individual relationships, visible on the RDA diagram (Fig. 1a, b), in the majority of cases correspond to the results of Spearman’s rank correlation test (Table 2). The closest positive relationships were observed between the air temperature and concentrations of *Cladosporium* and *Alternaria* spores in the hourly data set (*r*_s_ = 0.511 and *r*_s_ = 0.359, respectively), and *Cladosporium*, *Ganoderma*, *Drechslera* type and *Alternaria* spores in the daily data set (*r*_s_ = 0.601, *r*_s_ = 0.529, *r*_s_ = 0.525 and *r*_s_ = 0.480, respectively). An inversely proportional, statistically significant impact of the air temperature on the concentrations of spores in the hourly data set was observed for *Fusarium*, *Stachybotrys* and *Stemphylium*; however, all these negative correlations were very weak. The highest, but still weak, negative impact of the air temperature was observed in the daily data set for *Stachybotrys* spores (*r*_s_ = −0.215).

The second most important meteorological variable impacted fungal spore concentrations was relative humidity of the air. Following the gradient of increasing RH (Fig. 1), the maximum abundance of *Leptosphaeria* type, *Fusarium*, *Phaeosphaeria* and *Curvularia* spores was related to moderate values of RH in the hourly and daily data set, whereas the occurrence of *Ganoderma* spores differed in both data sets (in the hourly data set was associated with the moderate RH values, and in the daily data set was associated with the lowest RH values). The maximum abundance of all other spore types was observed at the lowest RH values in both data sets.

According to the results of Spearman’s rank correlation test (Table 2), all statistically significant and directly proportional relationships between RH and fungal spore concentrations were weak (*r*_s_ < 0.300) in both data sets. Negative impact of RH on the concentrations of spores was slightly stronger only for *Tilletia* and *Torula* in the daily data set (*r*_s_ = −0.350 and *r*_s_ = −0.400, respectively).

The last two meteorological variables, wind speed and precipitation, were statistically significant and explained the smallest amount of the total variance in the fungal spore composition (WIND 1–2%, PRECIP < 1%) (Table 5). However, distribution of individual fungal spore types along WIND gradients clearly differed between hourly and daily data set (was almost opposite).

According to the results of Spearman’s rank correlation test (Table 2), the highest, but still weak, negative impact of WIND on the concentration of spores was observed for *Ganoderma* (*r*_s_ = −0.325), and PRECIP—for *Tilletia* (*r*_s_ = −0.210) in the daily data set. Directly proportional, moderate impact of PRECIP was observed for *Leptosphaeria* type (*r*_s_ = 0.416) in the daily data set (Table 2).

## Discussion

Numerous studies on the effects of environmental factors on the presence of spores in the air are useful in developing predictive models and determining the direction of the spread of spores, what may be important, e.g., public health (allergenic spores), agriculture, horticulture and forestry (pathogens, plant disease control), aerobiology (particles suspended in the air). In this article, the authors present impact of selected meteorological parameters on the content and composition of the widest possible in this study spectrum of airborne fungal spores, in terms of hourly and daily values, for 1 year. Due to the time-consuming process, not many authors described the hourly concentration of spores in the air, and the models are created mainly for selected days and only for one or two genus or spore groups. In one of such studies, taking into account the hourly values of the variables in the days, when the *Alternaria* and *Cladosporium* spore concentrations exceed threshold values, Grinn-Gofroń and Strzelczak (2009) applied, among others, Spearman’s rank association test. Simple analysis revealed that relative humidity strongly and negatively influenced the concentration of *Alternaria* spores, and air temperature—strongly and positively (*r*_s_ = −0.657 and *r*_s_ = 0.582, respectively). Wind speed also significantly and positively influenced *Alternaria* spore concentration; however, it was weak correlation (*r*_s_ = 0.290). Precipitation was not a statistically significant variable. *Cladosporium* spore concentration correlated less strongly with relative humidity and temperature (*r*_s_ = −0.314 and *r*_s_ = 0.290), and not significantly with wind speed and precipitation. In our study, the hourly concentrations of *Alternaria* and *Cladosporium* spores were similarly positively correlated with air temperature; however, the relation was stronger with *Cladosporium* than with *Alternaria* (*r*_s_ = 0.511 and *r*_s_ = 0.359, respectively). As in the cited studies, relative humidity and precipitation negatively affect, and wind speed positively affects hourly concentrations of both types of spores, although the relationships were much weaker. In contrast, precipitation and wind speed significantly influenced the hourly concentrations of *Alternaria* and *Cladosporium* spores in our study (despite the fact that correlations are weaker, statistical significance is result of much higher number of samples).

The other similar study (Kasprzyk et al. 2011) showed that in the selected days, when the spore concentration exceeds threshold values, the relationships between hourly *Ganoderma* spore concentrations and meteorological variables differed in strength and direction between two cities located in two different climatic regions of Poland (maritime climate in Szczecin and continental climate in Rzeszów). In general, those dependencies were much weaker and mostly insignificant in Szczecin comparing to Rzeszów. Simple Spearman’s rank correlation analysis revealed in Szczecin directly proportional, weak influence of temperature on the hourly *Ganoderma* spore concentration (*r*_s_ = 0.010), as in our study (*r*_s_ = 0.179). However, in the cited study it was not statistically significant dependence. Conversely, hourly *Ganoderma* spore concentrations were negatively and significantly correlated with temperature in Rzeszów (*r*_s_ = −0.320). A weak positive and insignificant correlation with relative humidity was observed by Kasprzyk et al. (2011) in Szczecin (*r*_s_ = 0.030). Similarly, in our study it was a directly proportional, however statistically significant relationship (*r*_s_ = 0.183). Closest relationship with relative humidity was observed in Rzeszów in the cited study (*r*_s_ = 0.320). An inversely proportional impact was observed for wind speed—in Szczecin it was statistically insignificant (in our and cited studies), and in Rzeszów it was significant and weak (*r*_s_ = −0.190).

There are many articles on the influence of meteorological factors on the presence of spores in the air, taking into account daily values of variables and dependences between particular spore types and individual meteorological variables. Studies on the most allergenic spores of *Cladosporium*, *Alternaria*, *Drechslera* type, *Ganoderma* and *Epicoccum* revealed significant, positive correlation between daily mean spore concentration and daily mean air temperature (e.g., Troutt and Levetin 2001; Oliveira et al. 2009; Sadyś et al. 2015, 2016; Ianovici 2016; Ščevková et al. 2016). Generally, most airborne fungi are moderately temperature dependent—they tolerate neither too low nor too high temperature (Ingold 1971). In our study, simple analysis (Spearman’s rank correlation test) revealed the strongest, directly proportional influence of air temperature on the daily spore concentration of *Cladosporium*, *Ganoderma* and *Drechslera* type. Other significant relationships were moderate (*r*_s_ < 0.500 with *Alternaria*, *Polythrincium*, *Tilletia, Torula*) or weak (*r*_s_ < 0.300 with *Didymella*, *Pleospora*, *Chaetomium*, *Periconia* and *Epicoccum*). Daily concentration of *Stachybotrys* spore was the only variable significantly and negatively influenced by temperature, but this type of spores is very rare, and this relationship can be accidental.

Many studies revealed negative correlation between relative humidity and daily concentration of “dry-weather spore types” in the air. Stępalska and Wołek (2005), Grinn-Gofroń and Mika (2008), Oliveira et al. (2009), Sadyś et al. (2015, 2016), Ianovici (2016) found such significant dependences in case of *Cladosporium*, *Alternaria*, *Drechslera* type, *Epicoccum*, *Pithomyces*, *Polythrincium* and *Torula* spores. Our study confirmed significant, inversely proportional relationships between relative humidity and the concentration of spores: moderate with *Torula* and *Tilletia*, and weak with *Drechslera* type. Furthermore, simple analysis revealed in our study significant, directly proportional, weak impact of relative air humidity on the daily spore concentration of *Phaeosphaeria*, *Fusarium*, *Pleospora* and *Agrocybe*.

The direction of correlation between precipitation and spore concentration in the air depends on the spore discharge mechanisms and “rain-out and wash-out effects.” The strongest, directly proportional correlation with precipitation showed in our study the daily spore concentrations of *Leptosphaeria* type and *Stemphylium* (*r*_s_ = 0.416 and *r*_s_ = 0.300, respectively). Sadyś et al. (2015) found the spores of *Leptosphaeria* type the only group that was strongly linked to rainfall. Unlike in our study, in the Sadyś et al. (2015) analyses this resulted in a further close positive association with relative humidity. Grinn-Gofroń and Mika (2008) showed changeable dominance between rainfall and relative humidity in relation to *Leptosphaeria* type spore fluctuation.

Knowledge of wind statistics is important for modeling the aerial transport and surface-atmosphere exchange of fungal spores. Statistically significant results of our simple analyses showed only inversely proportional impact of wind speed on the daily spore concentration of *Ganoderma* (moderate correlation), *Chaetomium*, *Agrocybe* and *Epicoccum* (weak correlation). Hasnain (1993) also revealed significant, negative relationship between wind speed and *Ganoderma* spore concentration. In the study of Ianovici (2016), in general, the daily concentrations of *Alternaria, Cladosporium*, *Epicoccum* and *Pithomyces* spores were negatively correlated with wind speed. Most of these relationships were significant and weak.

The impact of complex of meteorological factors on the composition of spores has been analyzed in only a few studies, taking into account the daily values of the variables. Li and Kendrick (1994) applied canonical correspondence analysis (CCA—for unimodal data structure) to assess the relative effects of multiple environmental factors on the composition of twenty identified fungal genera and some groups of unidentified to genus spores. They took into account 16 environmental factors, including 6 meteorological variables. In a whole year, according to the arrow lengths (visible on the ordination diagram), the most important explanatory variables were found to be: relative air humidity, rain, vegetation, cloud, temperature and wind speed, in descending order. The authors did not assess the ranges of the explained variation of the spore composition and the statistical significance of individual environmental variables in explaining the fungal spore variation in the air (no results of stepwise forward selection). According to the ordination diagram (for the whole of 1992), spore concentration of *Leptosphaeria* type responded to high relative humidity and similar as the *Drechslera* type—increased during rain. Concentration of *Alternaria* spores decreased with rain and increased with temperature. Higher wind speed was positively related to the concentration of *Periconia* spores. CCA results (separate for February, May, August and December) revealed that the influence of some environmental factors on the airborne fungal spores varied with the season of the year.

In the next study on functional relationships of airborne fungi with meteorological factors, Li and Kendrick (1995) applied redundancy analysis (RDA—for linear data structure). For the whole of 1992, in accordance with the lengths of meteorological arrows (gradients), the ranking in descending order of importance was found to be: mean, minimum and maximum temperature, mean wind speed, relative humidity, rain, maximum wind speed and snow. Spore concentrations of most airborne fungal taxa were positively associated with air temperature. *Epicoccum* spore concentration was positively related with the wind speed. Spores of *Leptosphaeria* type showed a somewhat closer association with rain and relative humidity.

In our previous study (Grinn-Gofroń and Bosiacka 2015), we determined functional relationships between composition of airspora and meteorological factors using CCA (during a 4-year period and for each year separately). The CCA results indicated that all statistically significant variables accounted for 15.3% of the total variance in the spore data in the 4 years. The largest amount of the total variance was explained in this period by the mean air temperature (9.2%). In a recent published aerobiological study using an ordination method (RDA), Sadyś et al (2015) examined the daily relationship between 10 spore types and 8 meteorological variables (for 5 years separately). They determined which factors favored the most abundance of spores in the air, and moreover, applied variance inflation factor to indicate which explanatory variables were auto-correlated and needed to be excluded from analyses. The authors, similarly to Li and Kendrick (1994, 1995), did not use stepwise forward selection. Meteorological parameters were classified in the descending order of importance based solely on the length of the arrows, visible on the ordination diagrams. Repetitive tendencies in all years were in general: directly proportional impact of relative humidity and rain on the daily concentration of *Leptosphaeria* type and *Didymella* spores, and air temperature—on the daily concentration of *Alternaria* spores.

Our presented study indicates that temperature, relative humidity, wind speed and precipitation, in that order of importance, significantly influenced composition of airborne spora, both in hourly and daily periods of time. The range of explained total variance in the spore data was, in general, similar as in our previous study (Grinn-Gofroń and Bosiacka 2015). Air temperature had the strongest impact on the content and composition of spores—many times greater than the impact of other factors. In the ordination diagram, the same as in studies of Li and Kendrick (1994, 1995), Sadyś et al. (2015) and Grinn-Gofroń and Bosiacka (2015), spores of *Leptosphaeria* type were related to the highest or moderate values of relative humidity and precipitation. In contrast to the cited studies, spores of *Didymella* showed no closer association with these variables. As in the study of Grinn-Gofroń and Bosiacka (2015), the occurrence of *Cladosporium*, *Alternaria* and *Drechslera* type spores was associated with high temperature values. Such relationship between *Alternaria* spores and air temperature was also seen on the ordination diagrams in other cited studies (Li and Kendrick 1994, 1995; Sadyś et al. 2015), between *Cladosporium* spores and air temperature—in the study of Li and Kendrick (1995) and during some years—in the study of Sadyś et al. (2015). High, directly proportional impact of air temperature on the abundance of *Ganoderma* spores, visible in our presented RDA diagram (for daily data set), confirms the results achieved by Sadyś et al. (2015) in 1 of 5 years studied.

Basic meteorological parameters are useful in determining spore content and composition in the air, but the modeling of hourly data needs some further elaboration. Averaging the spore concentration into daily mean values smooths the relationship and enhances the performance of daily models. Therefore, hourly variations in spore abundance are much difficult to explain by meteorological parameters comparing to the overall daily sum of spores in the air. The results of Spearman’s rank association test and multivariate direct gradient analysis for hourly data in our presented study were of lower performance compared to these for daily data set. The primary reason is likely that the hourly spore concentrations fluctuate considerably, while most of the meteorological parameters remain rather stable in such a period of time. Key factors in hourly prediction of spore concentration and composition may be, for example, wind characteristics. The problem still demanding solutions in aerobiological studies is “isokinetic problem”: wind speed may fluctuate hour by hour, minute by minute, while the trap’ flow rate of air remains constant at a fixed speed resulting in two possibilities—sample speed exceeds air speed or air speed exceeds sample speed (Hirst 1952, 1953; Hasnain 1993). Further studies are needed to reveal additional parameters which could increase the accuracy of models for hourly spore contents.
